# Supplementary material for: A Meaningful Strategy for Glioma Diagnosis via Independent Determination of hsa_circ_0004214
Source: Brain Sci. 2023 Jan 23;13(2):193. doi: 10.3390/brainsci13020193 (PMC9954075; doi:10.3390/brainsci13020193)
Supplement: Supplementary file 1 [file brainsci-13-00193-s001.zip › File S1.pdf]

1. FISH-probe(hsa_circ_0004214)

**Name :**	**Fish probe**		
**Sequence(5'to3'):**	GTTCTTGGCGTGCTGACTGG		
**Lot No. :**	AX203112439	**Length :**	20
**Purification :**	HPLC	**Modification(5'to3'):**	5`Cy3
**nmoles:**	10.86	**Add water to 100uM:**	108.6
**TM(℃) :**	61.9	**GC(%) :**	60
**MW (target):**	6694	**MW(observed):**	6692.7
**Conclusion:**		**Qualified:**	
**Inspector:**		**Auditor:**	

FIG1

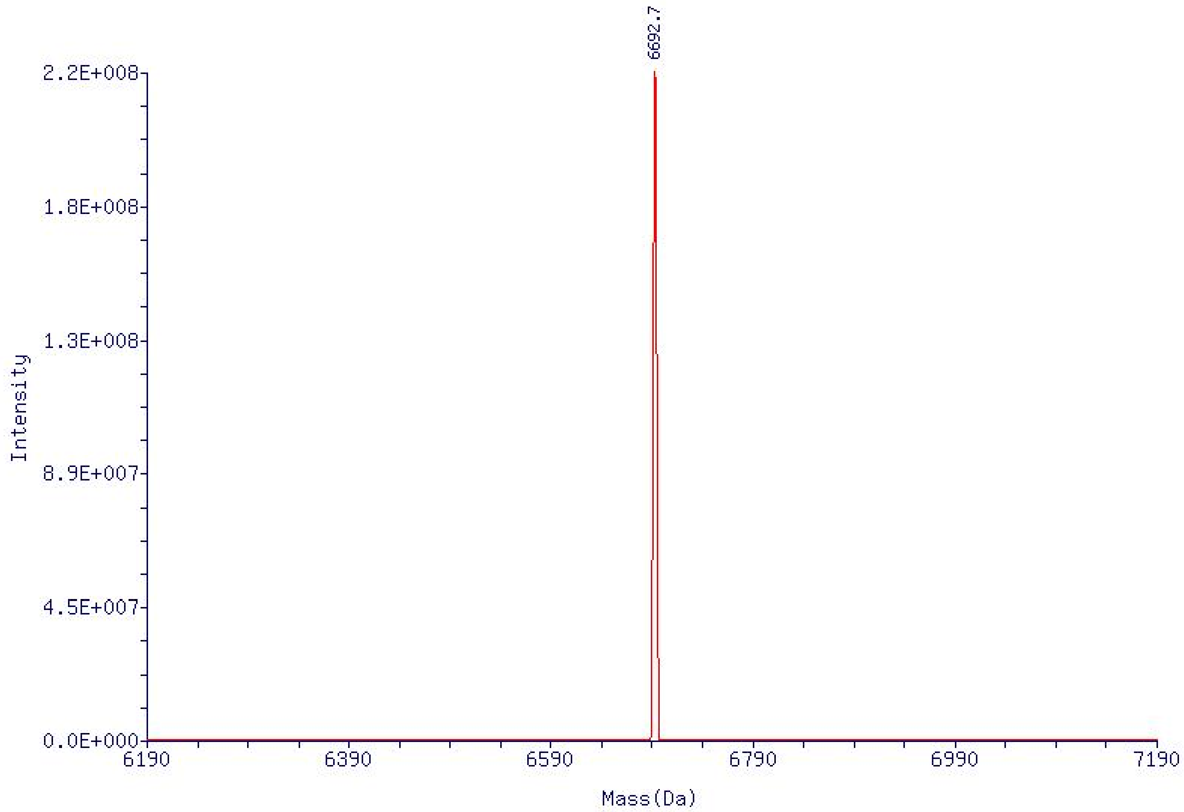

ESI - MS FIG1

2. CircAMOTL1 (hsa_circ_0004214) full-length gene.

TTGAAGATCCTCTTTGTAACTTCCACTCCCCAAACTTCCTGAGGATCTCAGAGGTGGAAATGAGAGGTTCCGAGGATGCGGCAGCTGGAA
CAGTATTGCAGCGGCTGATCCAGGAACAACTGCGGTATGGCACCCCAACCGAGAACATGAACTTGCTGGCCATTCAGCACCAGGCCACA
GGGAGTGCAGGACCAGCCCATCCTACAAACAACTTTTCTTCCACGGAAAACCTCACTCAAGAAGACCCACAAATGGTCTACCAGTCAGCA
CGCCAAGAACCGCAGGGTCAAGAACACCAGGTGGACAATACGGTGATGGAGAAACAGGTCCGGTCCACGCAGCCTCAGCAGAACAACG
AGGAACTGCCCACTTACGAGGAGGCCAAAGCACAGTCGCAGTTCTTCAGGGGGCAGCAGCAGCAGCAACAGCAGCAGGGGGCGGTGG
GCCATGGTTACTACATGGCAGGGGGCACCAGTCAGAAGTCCCGAACTGAGGGGAGGCCCACTGTGAACCGTGCCAACAGTGGACAGGC
GCATAAGGACGAGGCGCTGAAGGAACTGAAGCAGGGCCACGTCCGCTCGCTCAGCGAGAGAATCATGCAGCTGTCCCTGGAGAGGAAT
GGGGCCAAGCAACACCTTCCCGGCTCGGGGAATGGAAAGGGCTTCAAAGTAGGAGGGGGGCCCTCCCCTGCCCAGCCTGCAGGTAAA
GTGCTGGACCCTCGGGGTCCTCCACCTGAGTACCCCTTCAAGACCAAGCAAATGATGTCCCCAGTCAGCAAGACCCAGGAGCACGGACT
TTTTTATGGTGACCAGCACCCCGGGATGCTCCACGAGATGGTCAAGCCCTACCCTGCTCCTCAGCCTGTGAGAACAGATGTGGCCGTCCTG

CGGTACCAGCCACCCCCTGAGTATGGGGTAACGAG

3. Plasmid information. pcDNA3.1(+) CircRNA Mini Vector

FIG3

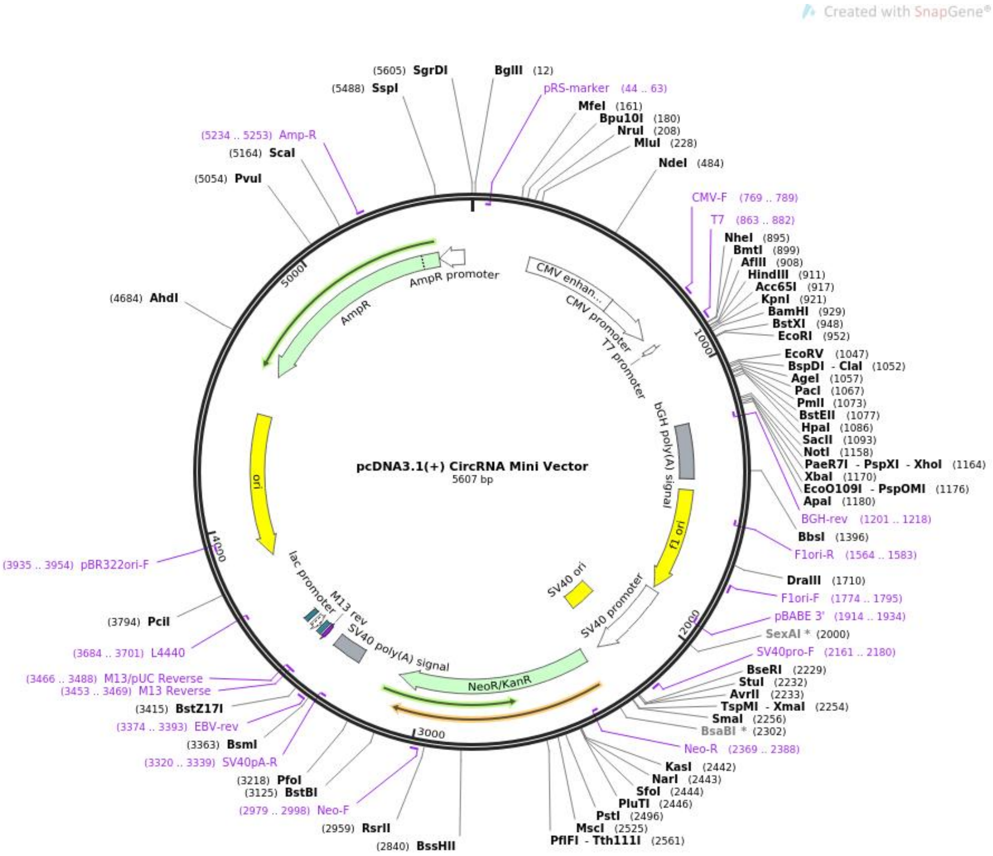

LOCUS pcDNA3.1(+) CircRNA Mini Vector 5607 bp ds-DNA circular SYN 10-JAN-2018

DEFINITION Expression plasmid for expressing circular RNAs of a desired

sequence.

ACCESSION .

VERSION .

KEYWORDS pcDNA3.1(+) CircRNA Mini Vector

SOURCE synthetic DNA construct

ORGANISM synthetic DNA construct

REFERENCE 1 (bases 1 to 5607)

AUTHORS Liang D, Wilusz JE

TITLE Short intronic repeat sequences facilitate circular RNA production.

JOURNAL Genes Dev. 2014 Oct 3. pii: gad.251926.114.

PUBMED 25281217

```
COMMENT    This file is created by Vector NTI
          http://www.biofeng.com/
COMMENT    ORIGDB|GenBank
COMMENT    VNTAUTHORNAME|biofeng.com|
COMMENT    VNTNAME|pcDNA3.1(+) CircRNA Mini Vector|
FEATURES             Location/Qualifiers
     source          1..5607
                     /organism="synthetic DNA construct"
                     /mol_type="other DNA"
     primer_bind     complement(44..63)
                     /label=pRS-marker
                     /note="pRS vectors, use to sequence yeast selectable
                     marker"
     enhancer        235..614
                     /label=CMV enhancer
                     /note="human cytomegalovirus immediate early enhancer"
     promoter        615..818
                     /label=CMV promoter
                     /note="human cytomegalovirus (CMV) immediate early
                     promoter"
     primer_bind     769..789
                     /label=CMV-F
                     /note="Human CMV immediate early promoter, forward primer"
     primer_bind     863..882
                     /label=T7
                     /note="T7 promoter, forward primer"
     promoter        863..881
                     /label=T7 promoter
                     /note="promoter for bacteriophage T7 RNA polymerase"
     primer_bind     complement(1201..1218)
```

```
             /label=BGH-rev
             /note="Bovine growth hormone terminator, reverse primer.
             Also called BGH reverse"
polyA_signal   1207..1431
             /label=bGH poly(A) signal
             /note="bovine growth hormone polyadenylation signal"
rep_origin     1477..1905
             /direction=RIGHT
             /label=f1 ori
             /note="f1 bacteriophage origin of replication; arrow
             indicates direction of (+) strand synthesis"
primer_bind    complement(1564..1583)
             /label=F1ori-R
             /note="F1 origin, reverse primer"
primer_bind    1774..1795
             /label=F1ori-F
             /note="F1 origin, forward primer"
primer_bind    complement(1914..1934)
             /label=pBABE 3'
             /note="SV40 enhancer, reverse primer for pBABE vectors"
promoter       1919..2248
             /label=SV40 promoter
             /note="SV40 enhancer and early promoter"
rep_origin     2099..2234
             /label=SV40 ori
             /note="SV40 origin of replication"
primer_bind    2161..2180
             /label=SV40pro-F
             /note="SV40 promoter/origin, forward primer"
CDS            2315..3109
```

```
                /codon_start=1
                /gene="aph(3')-II (or nptII)"
                /product="aminoglycoside phosphotransferase from Tn5"
                /label=NeoR/KanR
                /note="confers resistance to neomycin, kanamycin, and G418
                (Geneticin(R))"
                /translation="MIEQDGLHAGSPAAWVERLFGYDWAQQTIGCSDAAVFRLSAQGRP
                VLFVKTDLSGALNELQDEAARLSWLATTGVPCAAVLDVVTEAGRDWLLLGEVPGQDLLS
                SHLAPAEKVSIMADAMRRLHTLDPATCPFDHQAKHRIERARTRMEAGLVDQDDLDEEHQ
                GLAPAELFARLKARMPDGEDLVVTHGDACLPNIMVENGRFSGFIDCGRLGVADRYQDIA
                LATRDIAEELGGEWADRFLVLYGIAAPDSQRIAFYRLLDEFF"
primer_bind     complement(2369..2388)
                /label=Neo-R
                /note="Neomycin resistance gene, reverse primer"
primer_bind     2979..2998
                /label=Neo-F
                /note="Neomycin resistance gene, forward primer"
polyA_signal    3283..3404
                /label=SV40 poly(A) signal
                /note="SV40 polyadenylation signal"
primer_bind     complement(3320..3339)
                /label=SV40pA-R
                /note="SV40 polyA, reverse primer"
primer_bind     3374..3393
                /label=EBV-rev
                /note="SV40 polyA terminator, reverse primer"
primer_bind     complement(3453..3469)
                /label=M13 rev
                /note="common sequencing primer, one of multiple similar
                variants"
```

```
primer_bind    complement(3453..3469)
             /label=M13 Reverse
             /note="In lacZ gene. Also called M13-rev"
primer_bind    complement(3466..3488)
             /label=M13/pUC Reverse
             /note="In lacZ gene"
protein_bind   3477..3493
             /label=lac operator
             /bound_moiety="lac repressor encoded by lacI"
             /note="The lac repressor binds to the lac operator to
             inhibit transcription in E. coli. This inhibition can be
             relieved by adding lactose or
             isopropyl-beta-D-thiogalactopyranoside (IPTG)."
promoter       complement(3501..3531)
             /label=lac promoter
             /note="promoter for the E. coli lac operon"
protein_bind   3546..3567
             /label=CAP binding site
             /bound_moiety="E. coli catabolite activator protein"
             /note="CAP binding activates transcription in the presence
             of cAMP."
primer_bind    complement(3684..3701)
             /label=L4440
             /note="L4440 vector, forward primer"
rep_origin     complement(3855..4440)
             /direction=LEFT
             /label=ori
             /note="high-copy-number ColE1/pMB1/pBR322/pUC origin of
             replication"
primer_bind    complement(3935..3954)
```

```
                     /label=pBR322ori-F
                     /note="pBR322 origin, forward primer"
     CDS             complement(4611..5471)
                     /codon_start=1
                     /gene="bla"
                     /product="beta-lactamase"
                     /label=AmpR
                     /note="confers resistance to ampicillin, carbenicillin, and
                     related antibiotics"
                     /translation="MSIQHFRVALIPFFAAFCLPVFAHPETLVKVKDAEDQLGARVGYI
                     ELDLNSGKILESFRPEERFPMMSTFKVLLCGAVLSRIDAGQEQLGRRIHYSQNDLVEYS
                     PVTEKHLTDGMTVRELCSAAITMSDNTAANLLLTTIGGPKELTAFLHNMGDHVTRLDRW
                     EPELNEAIPNDERDTTMPVAMATTLRKLLTGELLTLASRQQLIDWMEADKVAGPLLRSA
                     LPAGWFIADKSGAGERGSRGIIAALGPDGKPSRIVVIYTTGSQATMDERNRQIAEIGAS
                     LIKHW"
     primer_bind     5234..5253
                     /label=Amp-R
                     /note="Ampicillin resistance gene, reverse primer"
     promoter        complement(5472..5576)
                     /gene="bla"
                     /label=AmpR promoter
ORIGIN
        1 gacggatcgg gagatctccc gatcccctat ggtgcactct cagtacaatc tgctctgatg
       61 ccgcatagtt aagccagtat ctgctccctg cttgtgtgtt ggaggtcgct gagtagtgcg
      121 cgagcaaaat ttaagctaca acaaggcaag gcttgaccga caattgcatg aagaatctgc
      181 ttagggttag gcgttttgcg ctgcttcgcg atgtacgggc cagatatacg cgttgacatt
      241 gattattgac tagttattaa tagtaatcaa ttacggggtc attagttcat agcccatata
      301 tggagttccg cgttacataa cttacggtaa atggcccgcc tggctgaccg cccaacgacc
      361 cccgcccatt gacgtcaata atgacgtatg ttcccatagt aacgccaata gggactttcc
      421 attgacgtca atgggtggag tatttacggt aaactgccca cttggcagta catcaagtgt
```

```
 481 atcatatgcc aagtacgccc cctattgacg tcaatgacgg taaatggccc gcctggcatt

 541 atgcccagta catgacctta tgggactttc ctacttggca gtacatctac gtattagtca

 601 tcgctattac catggtgatg cggttttggc agtacatcaa tgggcgtgga tagcggtttg

 661 actcacgggg atttccaagt ctccacccca ttgacgtcaa tgggagtttg ttttggcacc

 721 aaaatcaacg ggactttcca aaatgtcgta acaactccgc cccattgacg caaatgggcg

 781 gtaggcgtgt acggtgggag gtctatataa gcagagctct ctggctaact agagaaccca

 841 ctgcttactg gcttatcgaa attaatacga ctcactatag ggagacccaa gctggctagc

 901 gtttaaactt aagcttggta ccgagctcgg atccactagt ccagtgtggt ggaattcaaa

 961 gtgctgagat tacaggcgtg agccaccacc cccggcccac tttttgtaaa ggtacgtact

1021 aatgactttt tttttatact tcaggatatc atcgataccg gtttaattaa cacgtgggta

1081 accgttaacc cgcggaggta agaagcaagg aaaagaatta ggctcggcac ggtagctcac

1141 acctgtaatc ccagcagcgg ccgctcgagt ctagagggcc cgtttaaacc cgctgatcag

1201 cctcgactgt gccttctagt tgccagccat ctgttgtttg cccctccccc gtgccttcct

1261 tgaccctgga aggtgccact cccactgtcc tttcctaata aaatgaggaa attgcatcgc

1321 attgtctgag taggtgtcat tctattctgg ggggtggggt ggggcaggac agcaaggggg

1381 aggattggga agacaatagc aggcatgctg gggatgcggt gggctctatg gcttctgagg

1441 cggaaagaac cagctggggc tctagggggt atccccacgc gccctgtagc ggcgcattaa

1501 gcgcggcggg tgtggtggtt acgcgcagcg tgaccgctac acttgccagc gccctagcgc

1561 ccgctccttt cgctttcttc ccttcctttc tcgccacgtt cgccggcttt ccccgtcaag

1621 ctctaaatcg ggggctccct ttagggttcc gatttagtgc tttacggcac ctcgacccca

1681 aaaaacttga ttagggtgat ggttcacgta gtgggccatc gccctgatag acggtttttc

1741 gccctttgac gttggagtcc acgttcttta atagtggact cttgttccaa actggaacaa

1801 cactcaaccc tatctcggtc tattcttttg atttataagg gattttgccg atttcggcct

1861 attggttaaa aaatgagctg atttaacaaa aatttaacgc gaattaattc tgtggaatgt

1921 gtgtcagtta gggtgtggaa agtccccagg ctccccagca ggcagaagta tgcaaagcat

1981 gcatctcaat tagtcagcaa ccaggtgtgg aaagtcccca ggctccccag caggcagaag

2041 tatgcaaagc atgcatctca attagtcagc aaccatagtc ccgcccctaa ctccgcccat

2101 cccgccccta actccgccca gttccgccca ttctccgccc catggctgac taattttttt

2161 tatttatgca gaggccgagg ccgcctctgc ctctgagcta ttccagaagt agtgaggagg

2221 ctttttttgga ggcctaggct tttgcaaaaa gctcccggga gcttgtatat ccattttcgg
```

2281 atctgatcaa gagacaggat gaggatcgtt tcgcatgatt gaacaagatg gattgcacgc

2341 aggttctccg gccgcttggg tggagaggct attcggctat gactgggcac aacagacaat

2401 cggctgctct gatgccgccg tgttccggct gtcagcgcag gggcgcccgg ttctttttgt

2461 caagaccgac ctgtccggtg ccctgaatga actgcaggac gaggcagcgc ggctatcgtg

2521 gctggccacg acgggcgttc cttgcgcagc tgtgctcgac gttgtcactg aagcgggaag

2581 ggactggctg ctattgggcg aagtgccggg gcaggatctc ctgtcatctc accttgctcc

2641 tgccgagaaa gtatccatca tggctgatgc aatgcggcgg ctgcatacgc ttgatccggc

2701 tacctgccca ttcgaccacc aagcgaaaca tcgcatcgag cgagcacgta ctcggatgga

2761 agccggtctt gtcgatcagg atgatctgga cgaagagcat caggggctcg cgccagccga

2821 actgttcgcc aggctcaagg cgcgcatgcc cgacggcgag gatctcgtcg tgacccatgg

2881 cgatgcctgc ttgccgaata tcatggtgga aaatggccgc ttttctggat tcatcgactg

2941 tggccggctg ggtgtggcgg accgctatca ggacatagcg ttggctaccc gtgatattgc

3001 tgaagagctt ggcggcgaat gggctgaccg cttcctcgtg ctttacggta tcgccgctcc

3061 cgattcgcag cgcatcgcct tctatcgcct tcttgacgag ttcttctgag cgggactctg

3121 gggttcgaaa tgaccgacca agcgacgccc aacctgccat cacgagattt cgattccacc

3181 gccgccttct atgaaaggtt gggcttcgga atcgttttcc gggacgccgg ctggatgatc

3241 ctccagcgcg gggatctcat gctggagttc ttcgcccacc ccaacttgtt tattgcagct

3301 tataatggtt acaaataaag caatagcatc acaaatttca caaataaagc atttttttca

3361 ctgcattcta gttgtggttt gtccaaactc atcaatgtat cttatcatgt ctgtataccg

3421 tcgacctcta gctagagctt ggcgtaatca tggtcatagc tgtttcctgt gtgaaattgt

3481 tatccgctca caattccaca caacatacga gccggaagca taaagtgtaa agcctggggt

3541 gcctaatgag tgagctaact cacattaatt gcgttgcgct cactgcccgc tttccagtcg

3601 ggaaacctgt cgtgccagct gcattaatga atcggccaac gcgcggggag aggcggtttg

3661 cgtattgggc gctcttccgc ttcctcgctc actgactcgc tgcgctcggt cgttcggctg

3721 cggcgagcgg tatcagctca ctcaaaggcg gtaatacggt tatccacaga atcaggggat

3781 aacgcaggaa agaacatgtg agcaaaaggc cagcaaaagg ccaggaaccg taaaaaggcc

3841 gcgttgctgg cgtttttcca taggctccgc ccccctgacg agcatcacaa aaatcgacgc

3901 tcaagtcaga ggtggcgaaa cccgacagga ctataaagat accaggcgtt tccccctgga

3961 agctccctcg tgcgctctcc tgttccgacc ctgccgctta ccggatacct gtccgccttt

4021 ctcccttcgg gaagcgtggc gctttctcat agctcacgct gtaggtatct cagttcggtg

```
4081 taggtcgttc gctccaagct gggctgtgtg cacgaacccc ccgttcagcc cgaccgctgc

4141 gccttatccg gtaactatcg tcttgagtcc aacccggtaa gacacgactt atcgccactg

4201 gcagcagcca ctggtaacag gattagcaga gcgaggtatg taggcggtgc tacagagttc

4261 ttgaagtggt ggcctaacta cggctacact agaagaacag tatttggtat ctgcgctctg

4321 ctgaagccag ttaccttcgg aaaaagagtt ggtagctctt gatccggcaa acaaaccacc

4381 gctggtagcg gtttttttgt ttgcaagcag cagattacgc gcagaaaaaa aggatctcaa

4441 gaagatcctt tgatcttttc tacggggtct gacgctcagt ggaacgaaaa ctcacgttaa

4501 gggattttgg tcatgagatt atcaaaaagg atcttcacct agatcctttt aaattaaaaa

4561 tgaagtttta aatcaatcta aagtatatat gagtaaactt ggtctgacag ttaccaatgc

4621 ttaatcagtg aggcacctat ctcagcgatc tgtctatttc gttcatccat agttgcctga

4681 ctccccgtcg tgtagataac tacgatacgg gagggcttac catctggccc cagtgctgca

4741 atgataccgc gagacccacg ctcaccggct ccagatttat cagcaataaa ccagccagcc

4801 ggaagggccg agcgcagaag tggtcctgca actttatccg cctccatcca gtctattaat

4861 tgttgccggg aagctagagt aagtagttcg ccagttaata gtttgcgcaa cgttgttgcc

4921 attgctacag gcatcgtggt gtcacgctcg tcgtttggta tggcttcatt cagctccggt

4981 tcccaacgat caaggcgagt tacatgatcc cccatgttgt gcaaaaaagc ggttagctcc

5041 ttcggtcctc cgatcgttgt cagaagtaag ttggccgcag tgttatcact catggttatg

5101 gcagcactgc ataattctct tactgtcatg ccatccgtaa gatgcttttc tgtgactggt

5161 gagtactcaa ccaagtcatt ctgagaatag tgtatgcggc gaccgagttg ctcttgcccg

5221 gcgtcaatac gggataatac cgcgccacat agcagaactt taaaagtgct catcattgga

5281 aaacgttctt cggggcgaaa actctcaagg atcttaccgc tgttgagatc cagttcgatg

5341 taacccactc gtgcacccaa ctgatcttca gcatctttta ctttcaccag cgtttctggg

5401 tgagcaaaaa caggaaggca aaatgccgca aaaaagggaa taagggcgac acggaaatgt

5461 tgaatactca tactcttcct ttttcaatat tattgaagca tttatcaggg ttattgtctc

5521 atgagcggat acatatttga atgtatttag aaaaataaac aaataggggt tccgcgcaca

5581 tttccccgaa aagtgccacc tgacgtc
```

4 siRNA information.   **Custom Primers Information**

PIN A119404

Primer Name hsa_circ_0004214-siRNA-1-1907

Sequence(5’ to 3’): See Figure 4.

PIN A119405
Primer Name hsa_circ_0004214-siRNA-1-1907
Sequence(5’ to 3’): See Figure 4.

PIN A119406
Primer Name hsa_circ_0004214-siRNA-2-1493
Sequence(5’ to 3’): See Figure 4.

PIN A119407
Primer Name hsa_circ_0004214-siRNA-2-1493
Sequence(5’ to 3’): See Figure 4.

PIN A119710
Primer Name negative control.
Sequence(5’ to 3’): See Figure 4.

PIN A119711
Primer Name negative control.
Sequence(5’ to 3’): See Figure 4.

PIN A119712
Primer Name FAM negative control.
Sequence(5’ to 3’): See Figure 4.

PIN A119713
Primer Name FAM negative control.
Sequence(5’ to 3’): See Figure 4.

PIN A119714
Primer Name positive control (human GAPDH).
Sequence(5’ to 3’): See Figure 4.

PIN A119715
Primer Name positive control (human GAPDH).
Sequence(5’ to 3’): See Figure 4.

Custom Primers Information

Order NO.RX051061 Date:2022-03-21

Number of Primers:12 Number of Tubes:9

PIN	Primer Name	Sequence (5'to 3')	Length	Package	MW(g/mole)	Tm℃	GC%	nmol/tube	µg/tube	Volume for 100uM per tube/uL	Purification	Modification	Deliverables/tube
A119704	hsa_circ_0004214-siRNA-1-1907	/rG//rG//rA//rC//rU//rU//rG//rG//rC//rU//rG//rG//rA//rG//rA//rG//rA//rG//rA/TT	21	2.5 nmol*2	6828.25	54.4	52.4	2.5	17.1	25	HPLC	RNA	powder
A119705		/rU//rC//rU//rC//rU//rC//rU//rC//rC//rA//rG//rC//rC//rA//rA//rG//rU//rC//rC/TT	21		6501.96	54.4	52.4		16.3		HPLC	RNA	powder
A119706	hsa_circ_0004214-siRNA-2-1493	/rU//rG//rG//rA//rC//rA//rA//rG//rG//rC//rC//rA//rU//rG//rA//rG//rA//rA//rA/TT	21	2.5 nmol*2	6779.26	50.5	42.9	2.5	17	25	HPLC	RNA	powder
A119707		/rU//rU//rU//rC//rU//rC//rA//rU//rG//rG//rC//rC//rU//rU//rG//rU//rC//rC//rA/TT	21		6520.93	50.5	42.9		16.3		HPLC	RNA	powder
A119708	hsa_circ_0004214-siRNA-3-2748	/rC//rA//rA//rA//rG//rG//rG//rA//rC//rC//rG//rC//rA//rG//rA//rG//rA//rA//rA/TT	21	2.5 nmol*2	6801.31	52.4	47.6	2.5	17	25	HPLC	RNA	powder
A119709		/rU//rU//rU//rC//rU//rC//rU//rG//rC//rG//rG//rU//rC//rC//rC//rU//rU//rU//rG/TT	21		6513.89	52.4	47.6		16.3		HPLC	RNA	powder
A119710	阴性对照	/rU//rU//rC//rU//rC//rC//rG//rA//rA//rC//rG//rU//rG//rU//rC//rA//rC//rG//rU/TT	21	2.5 nmol*1	6583.01	52.4	47.6	2.5	16.5	25	HPLC	RNA	powder
A119711		/rA//rC//rG//rU//rG//rA//rC//rA//rC//rG//rU//rU//rC//rG//rG//rA//rG//rA//rA/TT	21		6732.19	52.4	47.6		16.8		HPLC	RNA	powder
A119712	FAM阴性对照	/rU//rU//rC//rU//rC//rC//rG//rA//rA//rC//rG//rU//rG//rU//rC//rA//rC//rG//rU/TT	21	2.5 nmol*1	7119.01	52.4	47.6	2.5	17.8	25	HPLC	5'6-FAM,RNA	powder
A119713		/rA//rC//rG//rU//rG//rA//rC//rA//rC//rG//rU//rU//rC//rG//rG//rA//rG//rA//rA/TT	21		6732.19	52.4	47.6		16.8		HPLC	RNA	powder
A119714	阳性对照（human GAPDH）	/rG//rU//rA//rU//rG//rA//rC//rA//rA//rC//rA//rG//rC//rC//rU//rC//rA//rA//rG/TT	21	2.5 nmol*1	6676.16	50.5	42.9	2.5	16.7	25	HPLC	RNA	powder
A119715		/rC//rU//rU//rG//rA//rG//rG//rC//rU//rG//rU//rU//rG//rU//rC//rA//rU//rA//rC/TT	21		6624.03	50.5	42.9		16.6		HPLC	RNA	powder

FIG 4

5. FISH Steps.

Fish detection experimental materials and instruments:

Reagents included 4% paraformaldehyde (Sigma, MKCL5723), 1% Triton x-100 (Biofroxx, 1139ML500), wet box, pre-hybridization solution (BOSTER, AR0152), oligonucleotide probe diluent (BOSTER, AR0062), DAPI staining solution (Beyotime C1005) and anti-fluorescence quenching mounting medium (Beyotime, P0126). The Fish probe sequence of hsa_circ_0004214 (5' to 3') is: GTTCTTGGCGTGCTGACTGG.

Experimental procedure:

1. The cells were placed at the bottom of the 24-well plate, so that they would be around 60%–70% confluent at the time of harvesting.

2. The cells were fixed with 1 mL 4% paraformaldehyde for 30 min and washed three times with PBS.

3. 0.4% Triton X-100 (diluted in PBS) permeable at room temperature for 15 min; Soak and wash PBS for 3 times, 3 min each time.

4. Wash with 2×SSC three times for 5 min each time. And dry at room temperature for 2-3 min.

5. Incubate in the pre-hybridization solution at 42°C for 30 min.

6. Dilute the probe concentration to 500 nM and denature at 85°C for 5 min.

7. Adding it to the specimen area of the slide under a cover slip, putting it in a humid chamber and hybridizing overnight at 42°C.

8. Washing twice at room temperature with 2×SSC, 5 min each time; washing twice at room temperature with 1× SSC, 5 min each time; washing twice at room temperature with 0.5×SSC, 5 min each time; washing with 0.1×SSC at room temperature.

9. The samples were re-dyed with 5μl DAPI and cleaned with PBS 10 min later.

10. The slides were mounted with anti-fluorescence quenching mounting solution and observed under a confocal fluorescence microscope.
