# Supplementary material for: A Meaningful Strategy for Glioma Diagnosis via Independent Determination of hsa_circ_0004214
Source: Brain Sci. 2023 Jan 23;13(2):193. doi: 10.3390/brainsci13020193 (PMC9954075; doi:10.3390/brainsci13020193)
Supplement: Supplementary file 1 [file brainsci-13-00193-s001.zip › File S2.pdf]

^DATABASE = GeoMiame
!Database_name = Gene Expression Omnibus (GEO)
!Database_institute = NCBI NLM NIH
!Database_web_link = http://www.ncbi.nlm.nih.gov/geo
!Database_email = geo@ncbi.nlm.nih.gov
^SERIES = GSE13030
!Series_title = Human primary and secondary glioblastoma vs. non-neoplastic adult brain tissue
!Series_geo_accession = GSE13030
!Series_status = Public on Dec 19 2009
!Series_submission_date = Oct 02 2008
!Series_last_update_date = Mar 20 2012
!Series_summary = MicroRNAs (miRNAs) are small (21-25 nucleotide in length) non-coding RNA molecules that negatively regulate protein expression. They are linked to cancer development and maintenance. In this work, studying gene expression profiles of 340 mammalian miRNAs with DNA microarrays, we selected 10 miRNAs gene features able to distinguish primary from secondary glioblastoma type; furthermore we verified that miR-21 and miR-155 up-regulatation seems to characterize the glioblastoma tumour state since it was found up-regulated in all samples analyzed compared to adult brain noneoplastic tissue. Since miR-21 function in glioblastoma cells was addressed previously we concentrated our efforts on miR-155 function. We found that miR-155 levels were markedly elevated both in primary and secondary glioblastomas tumours, in glioblastoma cell cultures and in 4 glioblastoma cell lines (U87, A172, LN229, and LN308) compared with adult brain tissue, CHP212-neuroblastoma cell lines and DAOY-1-medulloblastoma cell line. Since one of the miR-155 target was gamma-aminobutyric acid (GABA) A receptor (GABRA1) we verified if there was a relation between miR-155 up-regulation and GABRA1 expression. We demonstrated that, in cultured glioblastoma cells, knockdown of miR-155, which lower miR-155 expression to normal level, restore the normal expression of the gamma-aminobutyric acid (GABA) A receptor (GABRA1), making glioblastoma cells responsive to GABA cell cycle inhibiting signals. Our data suggest that aberrantly over-expressed miR-155 contribute to the malignant phenotype of the glioblastoma cells, promoting their unlimited growth.
!Series_summary =
!Series_summary =
!Series_summary = Keywords: miRNA expression profile
!Series_overall_design = We studied the expression profiles of 340 miRNAs in 97 glioblastoma tissues, of which 66 were primary glioblastomas and 27 were secondary glioblastomas. We have 66 replicates of primary glioblastoma and 27 replicates of secondary glioblastoma, each hybridized with the respective adult non-neoplastic brain tissue as a control.
!Series_type = Non-coding RNA profiling by array
!Series_contributor = Oscar,F,D'Urso
!Series_contributor = Pietro,I,D'Urso
!Series_contributor = Pasqualino,,Ciappetta

For details, please download from the website.

Volcano map, using 10 samples.

miR-124a	-1.614425	0.254242189
miR-125b	1.44886	0.069575937
miR-7	-1.208666667	0.499517854
let-7g	0.20214	0.491262071
miR-16	0.24328	0.266913966

miR-29b	-1.746075	0.276324414
miR-99a	-2.34032	0.096939903
miR-92	-0.12644	0.493909147
miR-324-5p	-0.66408	0.020291275
miR-132	-0.3201	0.736566641
miR-146a	-0.24964	0.6350523
let-7c	0.58332	0.25215134
let-7b	-0.20726	0.438662031
miR-9	-0.59716	0.573666489
let-7a	0.03564	0.924602349
miR-136	0.3624	0.427175208
miR-130a	-0.12748	0.805705252
miR-26b	-0.25368	0.423021288
miR-28	0.61045	0.389017599
miR-142-3p	-2.01936	0.12397008
let-7e	-3.40448	0.021206114
miR-137	-1.5719	0.358514971
miR-320	0.06382	0.248702482
miR-101b	1.22775	0.013034311
let-7d	-2.43392	0.15708912
let-7f	0.1255	0.502292722
miR-100	0.56728	0.388200985
miR-103	-0.05942	0.7888796
miR-106a	0.13274	0.386532477
miR-107	-0.2516	0.344562844
miR-10a	-1.56282	0.191154485
miR-10b	-0.53578	0.31585443
miR-122a	0.32106	0.606609016
miR-126	0.13672	0.596159454
miR-128a	-4.90774	1.75997E-06
miR-128b	-4.13582	0.022796077
miR-130b	-0.009866667	0.984239198
miR-133a	0.03938	0.924186412
miR-134	-0.155066667	0.833229172
miR-135a	-0.4487	0.443686669
miR-140	0.089	0.561998266
miR-141	-0.31085	0.621603471
miR-142-5p	-0.25738	0.870611603
miR-143	-3.57114	0.004574374
miR-144	-0.1152	0.341813371
miR-145	-1.32044	0.126338501
miR-148a	0.14078	0.896925434
miR-148b	0.351675	0.422325706
miR-149	-1.26934	0.027775557
miR-150	-2.49825	0.061747852
miR-152	-1.4493	0.403403277
miR-153	0.332875	0.535555632
miR-154	0.50045	0.503667867
miR-155	-0.33212	0.432284514

miR-15a	0.06104	0.466141208
miR-15b	-0.29824	0.488086813
miR-17-5p	-0.4712	0.032882093
miR-18a	-0.34356	0.486994979
miR-181a	-5.06348	6.56518E-07
miR-181b	-3.48926	0.076949166
miR-181c	-5.55144	1.01945E-05
miR-182	-3.293175	0.060467739
miR-183	-4.3128	0.020544943
miR-187	-1.398825	0.355182837
miR-192	-0.54482	0.237415456
miR-196a	0.7131	0.012115957
miR-199a	-0.80762	0.569201179
miR-199b	-1.03288	0.458438704
miR-19a	0.5752	3.51198E-07
miR-19b	0.49496	0.050484557
miR-20a	-0.3126	0.073150593
miR-203	-1.01496	0.474787926
miR-204	-1.28335	0.310874243
miR-205	0.116433333	0.90261553
miR-21	6.9285	1.8422E-08
miR-210	0	0
miR-211	0.7645	0.077695337
miR-212	-2.20714	0.218913573
miR-213	0.13114	0.935086089
miR-214	-1.3726	0.43123988
miR-215	-1.8924	0.192887115
miR-216	-2.39896	0.160038618
miR-217	-1.391325	0.380093955
miR-218	-5.81476	2.79466E-05
miR-219	-1.28826	0.320113282
miR-22	0.01886	0.990389644
miR-220	0.662133333	0.286103354
miR-221	3.482	1.87351E-07
miR-222	-1.51514	0.238200775
miR-223	0.13168	0.756815901
miR-23a	-1.6602	0.249165702
miR-23b	-0.0022	0.990445729
miR-24	-3.35425	0.052199163
miR-26a	0.13156	0.527102043
miR-27b	-0.00256	0.994031917
miR-29a	-1.02348	0.144277533
miR-30b	-0.13452	0.753340361
miR-30c	-2.05106	0.260034374
miR-30d	-0.04342	0.897750398
miR-34a	-1.273325	0.394348828
miR-9	-0.84762	0.55046002
miR-96	-1.274525	0.391802004
miR-98	0.5362	0.418765939

miR-29c	-0.636675	0.383063068
miR-30a-3p	0.222966667	0.518767351
miR-217	-3.2008	0.014674326
miR-199b	-3.58784	0.063305764
miR-211	-1.5427	0.364383255
miR-10b	-2.66604	0.077069169
miR-184	0.02486	0.951477106
miR-186	-1.610333333	0.342199407
miR-188	-1.57552	0.213317764
miR-190	-1.2589	0.394536029
miR-193a	-1.248975	0.422566519
miR-194	-1.90485	0.236993085
miR-195	0.24212	0.01203475
miR-197	-1.96292	0.177303312
miR-198	-0.940533333	0.369781157
miR-199a	0.9817	0.013047916
miR-200a	-1.0833	0.474632821
miR-200b	-0.358725	0.83330356
miR-201	-3.640825	0.030049503
miR-202	-1.298466667	0.460631913
miR-206	-0.16212	0.74181957
miR-207	-2.62498	0.031546329
miR-208	-4.200533333	0.027769699
miR-101	-0.32578	0.652285805
miR-105	-1.00694	0.472444187
miR-106b	-0.28994	0.240224916
miR-126	0.1266	0.633721833
miR-127	-1.88218	0.157781454
miR-147	-0.75292	0.354438705
miR-155	2.41764	9.79424E-08
miR-17-3p	-2.67695	0.172320295
miR-182	-1.43965	0.318542181
miR-189	0.6122	0.275751854
miR-224	-1.06275	0.027825768
miR-25	-1.4955	0.242557922
miR-27a	-0.4821	0.07392113
miR-296	-1.420125	0.323187778
miR-299-5p	-1.81074	0.341344465
miR-301	0.49324	0.352360821
miR-302a	-0.5875	0.324962043
miR-30a-5p	0.88292	0.132763365
miR-30e-5p	0.49846	0.673276449
miR-31	-2.41915	0.238238403
miR-32	-0.01122	0.968956925
miR-34b	-1.43428	0.474214177
miR-34c	0.2503	0.262658002
miR-93	-0.29422	0.076437712
miR-95	-1.79144	0.229868319
let-7d	-0.16232	0.830225127

miR-106a	0.09532	0.500609344
miR-135b	-2.7532	0.144295047
miR-151	-2.1285	0.236705699
miR-17-3p	-2.157966667	0.191649819
miR-224	-3.06085	0.100123604
miR-290	-1.1232	0.488207625
miR-291-3p	0.336375	0.062453457
miR-291-5p	-1.324525	0.381234657
miR-292-3p	-1.031833333	0.577092722
miR-292-5p	0.40854	0.605470197
miR-293	-1.56884	0.224661058
miR-294	0.400825	0.381374776
miR-295	-1.64125	0.253115454
miR-297	-1.7345	0.191816784
miR-298	-1.04606	0.459128055
miR-300	-0.6937	0.496782507
miR-323	-4.1466	0.031835368
miR-324-3p	-1.749033333	0.293991968
miR-325	-0.37484	0.469287523
miR-326	-4.145075	0.006163099
miR-328	-4.346433333	0.018684338
miR-329	-0.0366	0.975115313
miR-331	0.1964	0.78321365
miR-337	0.66622	0.34528704
miR-338	-3.20098	0.126876625
miR-339	-3.188375	0.016529439
miR-340	-2.37506	0.172049006
miR-341	0.878875	0.019266654
miR-342	-0.51882	0.035461996
miR-344	-2.91825	0.112578033
miR-345	0.9668	0.065896806
miR-346	-0.31718	0.71748811
miR-34b	0.2993	0.272106304
miR-351	0.07825	0.748226767
miR-7b	-4.084066667	0.037066951
miR-20	0.4491	0.426737494
miR-327	1.243833333	1.98279E-05
miR-333	-0.45846	0.685708537
miR-335	0.10562	0.782769776
miR-336	0.77906	0.153693915
miR-344	0.41385	0.76183679
miR-346	0.38198	0.35034975
miR-347	-0.6111	0.697010578
miR-349	1.00838	0.073310371
miR-352	-0.04532	0.946161271
miR-7	-2.7693	0.062197343
miR-133b	0.42864	0.55541826
miR-151	0.518625	0.130385509
miR-196b	-0.34474	0.703083658

miR-302b	-0.15074	0.736702351
miR-302b	0.05054	0.974479581
miR-370	-0.8753	0.542559977
miR-371	-3.92705	0.012043576
miR-372	0.3357	0.489256935
miR-373	-1.66952	0.196179114
miR-373	1.16255	0.00298497
miR-374	0.48148	0.506080112
miR-215	0.81174	0.153148278
miR-409-3p	-3.838466667	0.00336567
miR-410	-1.96914	0.2863083
miR-376b	-0.224	1.06223E-05
miR-376a	0.498133333	0.350171789
miR-411	1.02194	0.027411579
miR-380-3p	-0.5463	0.73972352
miR-412	-1.842566667	0.272217943
miR-521	0.133675	0.886403948
	0.36908	0.129497574
	-0.16598	0.375684124
	-0.1613	0.075832545
	0.18418	0.300775375
	0.04284	0.784495495
	0.29306	0.249272832
	0.1504	0.550077754
	0.24374	0.282165676
	0.05326	0.859568424
	0.26238	0.007909036
	0.31604	0.263101828
	-0.06402	0.75217375
	-0.2311	0.536106066
	-0.1116	0.711738714
	0.11092	0.515925525
	0.58882	0.041793396
	-0.24766	0.316945832
	-0.07532	0.780192495
	0.00394	0.982858843
	0.28548	0.121137021
miR-330	0.6975	0.119447024
miR-322	-0.0452	0.914730851
miR-424	-1.171675	0.492033153
miR-129-3p	0.7691	0.001199869
miR-361	-1.9106	0.15934215
miR-375	-1.2097	0.496768144
miR-377	-2.070733333	0.188206205
miR-378	1.49564	0.01019641
miR-379	-3.848825	0.016686089
miR-380-5p	-0.42716	0.689155301
miR-381	-0.03544	0.947817125
miR-382	-5.10646	0.005079175

miR-383	-3.53866	0.070384496
miR-384	-4.3031	0.021106578
miR-425	-0.00185	0.996213956
miR-452	-1.716525	0.249094643
miR-30e-3p	-1.283175	0.386329284
miR-362	-0.2636	0
miR-423	-1.15722	0.399415103
miR-18b	-5.90494	1.29564E-07
miR-20b	-0.05684	0.856792291
miR-363	0.37854	0.40104792
hsa-mir-520c, hsa-mir-520b, hsa-mir-520f	-0.96825	0.558658968
miR-200a	0.375066667	0.239242108
miR-302a	0.26274	0.642713
miR-299-3p	0.93464	0.141851409
miR-191	-0.8299	0.071096874
miR-154	-2.36032	0.079399351
miR-522	0.518766667	0.391021793
miR-369-5p	-1.639933333	0.233849451
miR-297	1.3736	0.006895184
miR-421	-1.535375	0.038857209
miR-463	0.72988	0.000962497
miR-464	-2.6327	0.170560947
miR-465	0.083775	0.872460688
miR-466	-0.32362	0.502362878
miR-467	-2.73498	0.120639266
miR-468	0.29314	0.301491737
miR-469	-1.2382	0.377396532
miR-470	-1.7519	0.209626461
miR-471	-0.1674	0.604772906
miR-434-5p	-0.09904	0.852787699
miR-434-3p	0.03812	0.955107603
miR-433-5p	-0.544633333	0.313815861
miR-129	0.052625	0.932327783
let-7i	0.44066	0.55345516
miR-200c	-0.58144	0.565491605
miR-350	-1.58025	0.263560024
miR-343	0.29738	0.674285432
miR-185	-4.805833333	0.003986106
miR-138	-1.010175	0.529571912
miR-191	-0.1423	0.578252556
miR-139	1.6145	0.007507379
miR-33	0.23855	0.666940734
hsa-mir-342	-0.28978	0.728000053
hsa-mir-19a	0.81164	0.001997053
miR-517a, miR-517b	-1.19788	0.406189574
miR-516-5p	-2.920975	0.11950435
miR-518b	-1.84986	0.218730735
miR-519d	-3.176275	0.084225448
miR-495	-0.9655	0.399633127

hsa-mir-28	0.0582	0.945640061
hsa-mir-519a-2	0.0844	0.875125939
hsa-mir-26b	-5.08335	0.000246813
miR-526c	-0.94936	0.516497397
miR-527	-3.96414	0.032262712
hsa-mir-29b-2	-5.34972	0.001328987
let-7g	-0.35465	0.593384591
miR-518a	-0.372233333	0.583940833
miR-523	-1.89646	0.16631804
miR-515-3p	0.7298	0.014902694
hsa-mir-146b	1.3505	
hsa-mir-222	-1.56052	0.276437325
miR-451	0.16176	0.444284866
hsa-mir-24	-3.0539	0.090207603
miR-503	0.3089	0.565398946
hsa-mir-505	-1.63048	0.402664255
miR-518e	0.57356	0.177461964
miR-432	-6.6439	
hsa-mir-193	-1.04785	0.472442438
miR-519a	-1.853625	0.180387748
miR-515-5p	-1.82368	0.337391685
hsa-mir-516-1	-1.943975	0.155723374
miR-140	-0.1936	0.5257002
hr-mir-151	-0.09422	0.52989588
hsa-mir-18	0.41935	0.666251539
hsa-mir-19b-1	-3.67405	0.024450982
hsa-mir-23a	-2.661	0.164159796
mir-527	-0.484575	0.465794553
hsa-mir-339	0.6523	0.006832283
hsa-mir-423	-0.07858	0.773889167
hsa-mir-425	-0.40536	0.601176143
hsa-mir-92-1	-1.862733333	0.252246966
hsa-mir-30d	-2.0677	0.11498807
miR-99b	0.16876	0.293126544
miR-433	0.916825	0.175973334
miR-431	-2.74785	0.154826435
miR-365	-0.91894	0.535583203
miR-450	-3.0131	0.017052082
miR-449	-1.1343	0.450439469
miR-429	0.28976	0.707793562
miR-448	0.014675	0.98565578

heat map, using 10 samples.

ID	1	2	3	4	5	6	7	8	9	10	
miR-18b	-4.4707	-6.6439	-6.6439	-6.5064	-5.2598	-6.6439					-58.7242
miR-181a	-4.1078	-4.3659	-6.6439	-4.7065	-5.4933	-4.4675					-51.2997
miR-128a	-3.5907	-6.6439	-5.088	-4.7293	-4.4868	-4.4422	-4.1714	-4	-4.7959	-3.6153	-45.5635
miR-181c	-6.6439	-3.2793	-4.5462	-6.6439	-6.6439	-3.1584	-2.7274	-3.3868	-3.399	-3.2109	-43.6397
miR-30c	0.2104	1.7225	-6.6439	-6.6439	1.0996	-6.6439	-4.2163	-6.6439	-6.6439	-2.0291	-36.4324
hsa-mir-29b-2	-6.6439	-6.6439	-6.6439	-0.173	-6.6439	-6.6439	0.8875	-6.6439	1.8875	1.9635	-35.2979
miR-469	1.1699	-1.5023	-0.881	1.6663	-6.6439	-6.6439	-6.6439	-6.6439	-6.6439	0.1137	-32.6529
miR-328	-6.6439	-6.6439		0.2485		-6.6439	-6.6439	-6.6439		0.4854	-32.4856
miR-527	-6.6439	-6.6439	1.848	-1.737	-6.6439	-1.4779	-6.6439			-3.8489	-31.7914
miR-384	-6.6439			-6.6439	0.3785	-0.1976	-6.6439	-6.6439	-6.6439	1.3785	-31.6601
miR-326	-2.041	-6.6439		-1.2515	-6.6439		-6.6439	-6.6439	-0.5353	-0.3659	-30.7693
miR-519d	-6.6439		-1.0954	-6.6439	1.6781	-6.6439	0.648	-6.6439	-6.6439	1.4594	-30.5294
miR-382	1.0433	-6.6439	-6.6439	-6.6439	-6.6439	-6.6439		1	-0.7084	1.9844	-29.9002
miR-371	-1.0985		-1.3219	-6.6439	-6.6439	0.9313		-1.2076	-6.6439	-6.6439	-29.2723
miR-181b	0.848	-6.6439	1.6374	-6.6439	-6.6439	-2.889	-6.6439	2.121	-3.9546		-28.8128
miR-132	-3.2243	-0.1472	1.8278	1.926	-1.9828	-6.6439	-6.6439	-6.6439	0.1257	-6.6439	-28.0504
miR-199b	0.5331	-6.6439	-6.6439	-6.6439	1.4594		-0.4739	-6.6439	-0.462	-1.6574	-27.1764
miR-379	-6.6439	-0.2345	-1.873	-6.6439			0.2203	-6.6439	1.585	-6.6439	-26.8778
hsa-mir-26b	-6.6439				-3.5228	-6.6439	-2.5479	-6.6439		-0.527	-26.5294
miR-383	-6.6439	-6.6439	0.538	-6.6439	1.7004	0.3219	-6.6439			-1.8943	-25.9096
miR-208		0.6862		-6.6439	-6.6439		-6.6439	1.8278	-6.6439	-1.1714	-25.233
miR-185	-6.6439			-6.6439	-1.1297	-6.6439	-1.0145		-3.0954		-25.1713
miR-218	-2.4982	-6.6439	-6.6439	-6.6439	-6.6439		1.9449	0.2726	1.8156	0.6304	-24.4103
miR-467	-1.4228	-6.6439	-1.6082	-6.6439	2.6439	-6.6439	0.6654	-6.6439	1.0108	1.0136	-24.2729
miR-143	-4.9885	-6.6439	-3.2793	-2.458	-0.486	-6.6439		0.2497	-0.2481	0.9419	-23.5561
miR-96	0.8074	1.1043		-6.6439	-0.3659	1.4854	-1.9714	-6.6439	-6.6439	-3.8079	-22.6798
miR-199a	1.433	-6.6439	1.6088	0.1375	-0.5735	-6.6439	1.893	-0.4521	-6.6439	-6.6439	-22.5289
miR-431		0.6627	-6.6439	1.6337	-6.6439	0.9686	-0.9351	-6.6439	2.0531	-6.6439	-22.1926
miR-450	-0.9241	-4.5031	-6.6439	-2.4422	-0.5522	-0.7934	0.9046	-0.6038	-6.6439	0.2054	-21.9966
miR-151	1.7004	0.1375	-6.6439	0.8074	-6.6439	-6.6439	2.0704	2.0531	-6.6439	-1.5187	-21.3255
miR-339		-0.3566	-3.9062	-6.6439	-1.8468	-2.2379	-6.6439			0.3219	-21.3134
miR-207	-2.9546	0.3265	-2.2447	-1.6082	-6.6439	0.2228	-1.9658	-2.4266	-0.1094	-3.673	-21.0769
miR-201	-6.6439	-6.6439	-1.6804		0.4049	-6.6439	0.6415		0.5341	-0.8034	-20.835
hsa-mir-516-1	-1.1016		0.5028	-0.5332	-6.6439	-6.6439	0.7433	-6.6439		0.3874	-19.933
miR-154	-6.6439	-0.5973	0.6323	-4.088	-1.1047	2.4463	-6.6439	-0.1633	-2.5649	-1.1811	-19.9085
miR-210						-6.6439	0.1375	-6.6439		-6.6439	-19.7942
miR-340	-6.6439	1.1043	-6.6439	1.0356	-0.7274	0.9804	-6.6439	-3.8868	-0.6506	2.7225	-19.3537
miR-425		-1.1552	1.1204	-0.3511	0.3785	1.3505	-6.6439	-6.6439	-1.458	-5.5873	-18.99
miR-137			-6.6439	0.184	1.7442	0.0426	-6.6439	-0.5778	-0.3827	-6.6439	-18.9214
miR-217	-1.5564		-6.6439	-3.9062	-0.6967	1.5361		-6.6439	-0.9159		-18.8269
miR-7b		1.0356		-6.6439	-6.6439		0.144		-6.6439		-18.7521
miR-128b	-2.458	-6.6439	1.7106	-6.6439	-6.6439	-0.8783	0.2179	0.926	1.211	0.4875	-18.715
miR-216	0.433	-6.6439	-6.6439	0.7225	0.1375	0.3219	-6.6439				-18.3168

miR-518a	0.848			0.4854	-2.4501		-6.6439	-4.3219	0.7467	-6.6439	-17.9797
miR-30a-3p		-0.8339		0.7655	0.7373		1.511	-6.6439	-6.6439	-6.6439	-17.7518
miR-211	0.6098	1.406			-6.6439	0.6781	-6.6439		-6.6439		-17.2378
hsa-mir-30d	-6.6439	0.1583	-1.3548	0.585	-3.0831		-6.6439	-2.0954	-0.255	2.511	-16.8218
miR-202	-6.6439		1.0704		1.6781	-0.3474	-6.6439		-6.6439	0.7655	-16.7651
miR-517a,miR-517b	-6.6439	-1.6394	-1	1.2558	2.0381	-6.6439	0.0704	-4.0589	2.5969	-2.3219	-16.3468
miR-298	-1.1266	1.5023	-0.5228	1.5607	-6.6439	-6.6439	2.3646	-0.0786	-0.0801	-6.6439	-16.3122
let-7e	-1.6758	-6.6439	-1.8678	-0.191	-6.6439	-0.4719	2.7655		-1.8468	0.4093	-16.1663
miR-24	0.107		-6.6439	-0.2362	-6.6439	2.1538		-6.6439		1.7442	-16.1629
miR-421	-0.2379	-1.7418	-0.4344	-3.7274		-2.8997	2.5361	-6.6439	-2.2042	-0.8034	-16.1566
miR-194	-3.2793	-6.6439	0.3219		1.9819	-1.4074	-1.5649	-6.6439	0.0704	1.3219	-15.8433
miR-297	-1.5105	-1.949	1.3785	0.0524	-6.6439	-2.2584	-0.1345	-0.7013	-3.1456	-0.8443	-15.7566
miR-183		0.3494	-6.6439	-6.6439		-6.6439	1.1454		1.406	1.6088	-15.4221
miR-99a	-0.1714	-6.6439	-0.1234	-0.0336	-4.7293	1.8532	0.6397	-0.6439	1.2491	-6.6439	-15.2474
miR-448	0.7655	-2.8262	0.1375		1.9819	-1.0559	-6.6439		-0.8599	-6.6439	-15.1449
miR-327		1.433	0.848		1.4505	1.585	-0.415	-6.6439	-6.6439	-6.6439	-15.0302
miR-34b	-6.6439	1.8679	2.0356	2.2129	-6.6439		1.1375	-1.9658	-6.6439		-14.6436
miR-31	-6.6439	1.2327		-6.6439	2.3785	-0.4266	-6.6439	0	2.2327		-14.5144
miR-338	-6.6439	-6.6439	-6.6439	1.9819	1.9449				0.4854	1.1375	-14.382
miR-470	-6.6439		-0.488	0.7216	-0.5973	1.0286	-0.3978	-4.5031	-4.1153	0.6781	-14.3171
miR-222	0.585	-6.6439	-0.482	0.3573	-1.3921	-1.2653	-1.5228	2.1043	-1.4659	-4.436	-14.1614
miR-293	0.4222	-1.8009	-6.6439	0.4542	-0.2758	2.278	-6.6439	0.848	-1.873	-0.8391	-14.0742
miR-141	0.6323	0.9635	-2.5064		-0.3328	-6.6439	1.9449	-6.6439	-1.4344		-14.0207
miR-10a	-1.1266	1.848	-1.1234	-5.8039	-1.6082	-6.6439	-1.077	0.2016	1.7004		-13.633
miR-105	2.1538	-0.2142	0.3045	-6.6439	-0.6349	2.0531	-6.6439	1.6554	-2.2243	-3.3076	-13.502
miR-212	1.6521	1.2016	-6.6439	-0.6016	-6.6439	1.1991	1.6781	0.7225	0.585	-6.6439	-13.4949
miR-149	-0.9743	-1.7084	-1.1714	0.4071	-2.8997	0.8448	-6.6439		-0.5291	-0.7588	-13.4337
miR-122a	1.8278	0.926	0.6425	0.3674	-2.1584	-6.6439		-0.6735	-6.6439	-1	-13.356
miR-292-3p	1	-6.6439		2.5484		-6.6439	-0.255	-4.662	1.4594		-13.197
miR-380-3p	1.3057	0.9358		-6.6439	2.2172	-6.6439	-3.7819		-0.2226	-0.3364	-13.17
miR-145	-3.3076	-3.4596	0.1177	0.6844	-0.6371	-6.6439	-0.8365	-0.4779	-0.6148	2.278	-12.8973
miR-432			-6.6439	-6.6439		0.7689	-0.5543	0.7022		-0.4699	-12.8409
miR-516-5p	-6.6439	-6.6439	0.1979	1.406		2.0356	0.7832	-6.6439	1.2928	1.5361	-12.6801
miR-150	0.0704	-2.5649	-6.6439	-0.8546		0.1674		-0.4719	1	-3.3629	-12.6604
hsa-mir-24	0.5361	-6.6439					-1.2379		1.4594	-6.6439	-12.5302
hsa-mir-19b-1		-0.6349	-6.6439	-6.6439	-0.7735	1.7874	0.7983	0.6323	-0.4679	-0.5228	-12.4689
miR-217	-1.4901		2.4983	0.0704	-6.6439	1.3127	-0.3997	-0.7298	-6.6439	-0.3827	-12.4087
miR-519a	0.0524	0.3516	-1.1746	-6.6439		-2.7084	1.1043	-0.5395	-0.6171	-2.1714	-12.3466
miR-204	-5.4235	-0.3076		-1.2898	1.8875	0.6654	1.8679	-6.6439		-3.0469	-12.2909
miR-452	-6.6439	1.8875		-1.0923	-1.0174	0.848		0.585	-6.6439		-12.077
miR-135b		-6.6439			1.1375	-1.4501	1.5607		-6.6439		-12.0397
hsa-mir-23a			-6.6439	1.3219				-6.6439			-11.9659
miR-322	0.5646	-1.7418	0.7824	-0.2042	0.373	1.6572	-1.1877	1.2029	-6.6439	-6.6439	-11.8414
miR-526c	-6.6439	1.7655	1.9069	-0.3603	-1.415			-2.1392	-2.0529	-2.5649	-11.5038
miR-344	0.8074		-6.6439			-6.6439	1.6088	-2.152		1.5607	-11.4629
miR-410	0.3219	1.8875	-6.6439	1.2327	-6.6439	-0.1313	0.2016	-0.1345	0.111	-1.6038	-11.4027
miR-409-3p	-2.035	-6.6439			-2.8365	2.278	2.3363	2.4463	-6.6439	-0.2226	-11.3213

miR-365	-0.5522	-6.6439	1.263	-1.2345	2.5729	-0.2009	0.8742	-0.1617	-6.6439	-0.48	-11.207
miR-361	-6.6439		-0.4941	-0.706	0.2016	-0.2126	-6.1078	1.4787	1.6215	-0.2396	-11.1022
miR-523	-6.6439	0.5361	-3.5228	0.2376	-0.0893	1.585		0.585	-2.3219	-1.458	-11.0922
miR-224	1.7225	-6.6439	-6.6439	-0.6781		-1.5864		1.7866		0.959	-11.0842
miR-464			-6.6439		1.3785	1.0356				-6.6439	-10.8737
miR-34b	-0.3733	1.0158	-0.1234		0.6781	1.7974	-6.6439	-6.6439		-0.454	-10.7472
hsa-mir-505	1	-6.6439	2.2479	-6.6439	1.8875	1.5607	-6.6439	2.0875	0.4147		-10.7334
miR-214	1.6781			-6.6439	0.848	0.3785	-2.482	-4.4933			-10.7146
miR-515-5p	-6.6439	0.2916	1.585	-6.6439	2.2928	-0.8783	-2.8997	2.3219	-0.097		-10.6715
miR-191	-1.8417	-2.0116	-0.1297	-0.1665	0	-2.2863	-0.8573	-0.0321	-5.3219	2	-10.6471
miR-9	2.848	-0.3292	-1.1456	-0.1266	-4.2324	-0.173	-2.9214	-3.0116	1.3039	-2.8468	-10.6347
miR-7	0.107	-6.6439	-1.771			-1.2793	-6.6439	2.406	1.4594	1.7866	-10.5791
miR-199b	-0.0831	0.3173	2.2327	-6.5328	-1.0985	1.511	0.8875	1.6554	-2.699	-6.6439	-10.4534
miR-370	-0.7735	0.4792	-6.6439	2.406	0.1557	-0.6259	0.5361	0.7225		-6.6439	-10.3877
miR-199a	0.2558	1.9635	0.4147		1.2928	0.0229		-1	-6.6439	-6.6439	-10.3381
miR-377		-6.6439		0.2798	0.1519	1.6201	-0.9686	-6.6439		2	-10.2046
miR-522		1.4854	-1.3076		1.3785	-6.6439	-0.3921	-0.2396	2.2016	-6.6439	-10.1616
miR-433-5p		1.0704	-1.4074		-1.2969		-0.6781	-6.6439		-1	-9.9559
miR-291-5p	1.7974	-6.6439	0.2265	-0.6781			-6.6439	1.3505	-1.1943	2	-9.7858
miR-140	-0.255				0.433	0.5281	-6.6439	1.3219	-3.4066	-1.7274	-9.7499
miR-95	-6.6439	-4.088	-0.3921	0.7883	1.3785	0.1966	0.4803	-1.4422		0.0552	-9.6673
hsa-mir-193	1.4431	-6.357	0.7225	0		-6.6439	-0.3585	1.7655	-0.725	0.5049	-9.6484
miR-23a	1.9866		-5.3808		-1.5864	-6.6439	1.406	1.7887	-1.0619	-0.1329	-9.6246
miR-376a	2		0.2314		-0.737	1.2516	-0.4921	-6.6439	-5.227		-9.617
miR-323	0.848		-6.6439	-6.6439		1.2588	0.2277	-0.5479	1.926		-9.5752
miR-34a	-6.6439		-0.0559	0.0704	1.5361	1.2928	-6.6439	0.2016	0.7655	-0.0336	-9.5109
hsa-mir-222	1.7442	0.4093	-6.6439	-3.0469	-0.2653	0.315	-1.5395		-0.1926	-0.1633	-9.383
miR-188	-6.6439	-1.3219	-0.3219	-0.1844	0.5945	1.1538	-4.088	0.7655	0.6781	0	-9.3682
miR-182		-0.4699	0.585	-6.6439	-6.6439	1		0.4967		2.3923	-9.2837
miR-29c	-0.5395	-0.6943	1.5236	-2.8365		-3.9214	0.2654	-1.5395	-1.4035	0.1296	-9.0161
miR-197	-3.5977	-0.5522	2.1538	-6.6439	-1.1746	0.1427	-0.6371	2.1538	-1.0619	0.2016	-9.0155
miR-423	-0.488	2	-6.6439	-0.5649	-0.0893	1.3785	-0.6552	0.8945	-6.6439	1.8278	-8.9844
miR-17-3p	-6.6439	1.9449		0.6351	-6.6439			-0.3529	-0.1714	2.3363	-8.8958
hsa-mir-18	0.9635	1.4854		-3.1078	2.3363	2.3923	-6.6439	-6.6439	-1.6259	2.121	-8.723
miR-147	2.1043	-0.1488	-1.2969	-3.0831	-1.3401	1.2141	-1.4422		-6.6439	1.926	-8.7106
miR-25	0.5907	-0.2793	-6.6439	0.1519	-1.2969	0.5588	1.8278	0.3629		-3.9613	-8.6893
miR-142-5p	-6.6439	2.0531	-0.9351	2.0531	2.1859	-0.3419	-2.6529	-2.4659	-1.0291	-0.897	-8.6737
miR-351				0.6304	-0.4739		-3.0954	-6.6439	2.0531	-1.0831	-8.6128
miR-349	0.8286	2.2928	-0.3978	2.2479	0.0704	0.9635	-5.5735	-4.161	-6.6439	2.0875	-8.2855
miR-449	0.585		1.1998	-6.6439	0.3219	1.585	1.433			-6.6439	-8.1631
miR-182	-6.6439	0.3173		0.7974	-0.2294	1.8797	-0.144	-6.6439	0.585	1.9635	-8.1183
miR-33	-0.9518	2.0179	-0.7442	0.6323		-1.7959		-0.55		-6.6439	-8.0356
miR-292-5p	-2.5995	1.511	-0.1031	2.2708	0.9635	-6.6439	0.2388	2	1.0704	-6.6439	-7.9359
miR-206	0.0524	-0.5842	1.2863	0.3346	-1.8997	0.0144	0.6397	-1.4659	0.3277	-6.6153	-7.91
let-7d	-6.6439	0.4447	-0.6484	-6.6439	1.3219		2.1043		0.4657	1.7442	-7.8554
miR-299-5p	-6.6439	2.121	1.8074	0.3057	-6.6439	-0.0589	-0.5929	0.848		1.1043	-7.7532
miR-101	-0.6038	-1.5951	2.585	-1.6804	-0.3346			-6.6439	0.0635	0.5361	-7.6732
miR-148b		0.5069	1.2054	0.8953	-1.2009	-6.6439	2	-0.2636	-6.6439	2.4725	-7.6722
miR-29b	-2.699	2.3329	-6.6439	0.0257		0.1661	-2.3884	-1.8943	2.263	1.2091	-7.6288

let-7d	-1.0469	1.0356	1.5361	-2.9214	0.585	-6.6439	0.6323	-1.0923	0.3573		-7.5582
miR-142-3p	-6.6439	-3.0831	0.8576	-0.7108	-0.5166	-0.0262	2.6323	-0.7784	0.4038	0.3774	-7.4879
miR-465	0.0158	-0.9406		-0.685	1.9449	-6.6439	0.9486	-0.482	-3.0831	1.585	-7.3403
miR-98		0.0731	-1.2619	2.6439	0.6897	0.4255	-2.6712		-0.486	-6.6439	-7.2308
miR-412			-6.6439	1.7004	-0.5842	1.8074	-0.0589	1.511	1.7004	-6.6439	-7.2117
miR-302b	-6.6439	0.5361	2.3074	2.0531	2	2.5729	0.2412	-3.6082	-6.6153	0.0704	-7.0863
miR-521	2.2928	-1.0439		-2.5821	1.8679	-6.2109		0.2154	-1.949	0.3265	-7.0833
miR-136	1.406	-0.0939	1.1375		-1	1.4998	1.3397	2.0179	-6.6439	-6.6439	-6.9808
miR-342	-0.4521	-1.2969	-0.2294	0.0786	-0.6943	-0.9771	-0.8186	-1.1361	-0.6148	-0.7539	-6.8946
miR-29a	-3.1714	-0.2447	-0.3511	-2.114	0.7638	0.3033	0.8758	-0.949	-1.3659	-0.5842	-6.8374
miR-127	-3.0469	-6.6439	0.263	0.6608	-0.6439	0.4103	-0.48	0.5548	1.2327	0.9396	-6.7535
miR-10b	-0.5187	-5.904	-6.6439	-0.2636	0	1.4657	0.3885	1.4854	2.406	1.0996	-6.485
miR-18a	-0.9105	0.2016	-0.7934	-1.6215	1.406		-0.4481	1	-6.6439	1.3357	-6.4741
miR-518b	0.3346	-4.2029	-6.6439	-0.4739	1.7368	1.4947	1.1699	-0.6349	-1.1488	1.9449	-6.4235
miR-154	0.544		-2.0529	1.2327	2.278		-1.458	-1.2176	0.9176	-6.6439	-6.4001
miR-424		-2.2934	2.0179	-6.6439	2.2327	-1	0.9635	-1.6574	0.0496		-6.331
miR-146a	1.4152	-1.9714	0.269	-0.8186	-0.1424	-0.4481	-1.6394	-2.041	-0.8916	0.1427	-6.1256
miR-369-5p		-0.5995		-5.553	1.2327		0.1375		0.144	-1.4501	-6.0884
miR-205	1.4854	1.7004	-2.8365			-0.1943	-0.0695		-6.6439	0.6304	-5.928
miR-376b			-0.2636		-0.1844	-1.191	1.9449	-0.1649	0.5753	-6.6439	-5.9276
hsa-mir-146b	1.3505					1.8875	-2.4035	0.543	-6.6439	-0.5312	-5.7976
miR-324-3p	-0.0233	1.4201	-6.6439			-6.6439	0.9015	2.406	1.6471	1.2016	-5.7348
let-7g		-1.8468	1.1375			-1	0.7655	-6.6439	1.433	0.6323	-5.5224
hsa-mir-342	1.4854	-2.2793	1.2928	-2.6259	0.6781	1.5361		-6.6439	1.2016		-5.3551
miR-296	0.3785		-6.6439	0.3219	0.263		-0.9269	1.3505			-5.2569
hsa-mir-28		2		-1.8836			-0.3474	-6.6439	1.7004		-5.1745
miR-347	-1.1361	-6.6439	3.009	2	-0.2845	1.2191	1.4297	1.5059	-6.0209	-0.1811	-5.1028
miR-148a	2.6893	0.2016	-3.9258	2.1538	-0.415		0.0482	-6.6439		0.848	-5.0438
miR-495			-3.4917	1.5607			1.8074	1.9449		-6.6439	-4.8226
miR-203	1.3505	-6.6439	-0.454	1.7866	-1.114	2.6893	-0.5907	-1.1297	-0.0847	-0.5543	-4.7449
miR-331	-2.3004	1.0704	1.7866		0.229	-0.9024	-6.6439		0.9811	1.0854	-4.6942
miR-34c		0.7424	-0.3789	0.3874			-0.2671	1.4854	-6.6439		-4.6747
miR-153	-0.5713	2.2327		-0.5929	0.263			-6.6439	-0.4093	1.159	-4.5627
miR-300	0.0398	1.5632	-3.6841			-0.816			-1.6394		-4.5365
miR-434-3p	-1.5951	-1.5951	1.2732	1.9819	0.1257	0.9635		0.8074	0.1776	-6.6439	-4.5048
miR-518e	-0.0425	1.4641	-0.274	-0.0664	1.7866	-0.7637	1.9635	-1.8783	-6.6439		-4.4546
miR-189	-0.685	-0.035		0.7225	2.4463	-6.6439	-3.7122	2.4463	2.5729	-1.415	-4.3031
miR-152	1.7983		0.4977	-6.6439		0.433	-0.9078	0.6572		-0.038	-4.2035
miR-372	0.543	0.1648	0.03	-1.1297	2.0704	1.8172	-2.0291	-4.3511	-2.0233	0.7502	-4.1576
miR-124a	-0.0116	-6.6439		-0.5395	0.7373	1.2054	0.1557		0.9426		-4.154
miR-93	-0.811	-0.4131	0.103	0.0029	-0.3529	-0.456	0.0607	-0.9856	-1.1424	-0.0247	-4.0191
miR-346	2.2327	1.3092	-1.221	-0.8599	-3.0469	-6.6439	0.7163	0.3973	1.5534	1.5607	-4.0021
miR-192	-2.2863	0.0786	0.189	-0.8599	0.1545	-1.7563	0.4233	-0.1424	0.451	-0.2142	-3.9627
miR-215		0.3022	-6.6439	-2.1584	0.9305	0.044	-0.4364	1.3494	1.6554	1	-3.9572
hr-mir-151	-0.1763	-0.0755	0.1852	-0.6506	0.2461	-1.7859	0.1545	-0.3921	-0.1795	-1.2758	-3.9499
miR-325	-1.6349	-0.3921	1.5484	-1.221	-0.1746	-0.3147	-0.0649	-0.692	-0.7346	-0.2653	-3.9457
miR-220		1.5724		-1.1844	1.5984	1.585	0.4308	1.4362	-6.6439	-2.699	-3.9045
hsa-mir-92-1	0.0922			0.9635	-6.6439	-1.8678	1.2271	1.263		1.2079	-3.758
miR-302a	-0.9771	-2.4422	-1.1297	0.6517	0.9598	0.8074	1.6631	0.4125	-0.6215	-2.8997	-3.5757

miR-373	-6.6439	-0.5929	-0.243	-1.8678	1	-0.0679	0.648	1.3505	1.923	0.926	-3.568
miR-219	-6.6439	0.0704	0	-0.6171	0.7493	0.0772	0.4854	2	-0.3456	0.6627	-3.5616
miR-193a	-6.6439	2.0875	0.5926	-1.0321		0.6323	0.3785		0.8711	-0.4403	-3.5543
miR-466	-1.2515	-0.9296	1.319	-1.1943	0.4383	-0.6967	-0.1031	-0.3752	-0.0634	-0.6282	-3.4847
let-7i	2.8875	0.1414	0.0676	-1.9942	1.101	-0.9686	-0.1746	0.9486	-2.4035	-3.035	-3.4298
miR-138	1.5748	1.9635	-6.6439		-0.9351	0.5607	-0.4305	1.9542	-2.3364	0.8711	-3.4216
miR-9	1.7004	1.263	0.2892	-0.8468	-6.6439	0.9753	0		0.8647	-0.852	-3.2501
miR-30e-3p	0.2016		-6.6439	1.2768	0.0328		-0.3789	0.8024	1.6088	-0.1456	-3.246
miR-106b	0.4647	0.1243	-0.6148	-0.9379	-0.486	-0.3659	-1.2793	-0.3978	0.9986	-0.7155	-3.2096
miR-129	-0.7442		1.3746	1.3219	-1.7418	1.4547	0.1375	1.6781	-6.6439	0	-3.1631
miR-324-5p	-1.191	-0.5778	-0.4739	-1.2515	0.1738	-1.7274	0.9381	-0.0365	-0.7346	1.748	-3.1328
miR-295	-6.6439		1.2016	-0.3566	-0.7661		0.4854	1.433	0.926	0.6378	-3.0828
miR-411	0	1.6554	0.0976	1.1551	2.2016	-0.2969		-6.6439	1.3907	-2.5146	-2.955
miR-503	-0.0499	2.1375		0.189	-1.041		2.0875	0.433	-6.6439		-2.8878
miR-20a	-0.3183	-0.8288	-0.4247	-0.2226	0.2314	0.0286	-0.1779	-0.0786	-0.9379	-0.0862	-2.815
miR-107	-0.2464	0.2963	-1.3147	0.0257	-0.0189	-1.3585	0.315	-0.5374	0.0172	0.0144	-2.8073
miR-433	-1.4344	1.3219	1.7442		2.0356	-6.6439			-0.3696	0.5597	-2.7865
miR-135a	-1.071	0.6763	-1.685	1.3757	-1.5395	-0.0262	-1.2109	0.3494	-0.486	1.0902	-2.527
miR-103	-0.0786	-0.1062	-0.4481	-0.4961	0.8319	-1.3219	-0.2142	0.5675	0.2987	-1.5228	-2.4898
miR-130b			1.4594	-1.1779	-0.3111	-0.1125	2.3363	2.5361	-6.6439	-0.527	-2.4406
miR-26b	-0.1203	-1.4035	0.6717	-0.3529	-0.0634	-0.9187	-1.3622	0.8584	0.848	-0.5821	-2.425
miR-463	0.8875	0.5529	1	0.1545	1.0545	1	-0.5395	0.1699		-6.6439	-2.3641
let-7b	0.0895	-0.1617	-0.1649	-1.2481	0.4489	-0.4521	-0.3529	-0.8625	-0.7612	1.1454	-2.3196
miR-333	-0.1568	1.2516	0.2004	1.5607	-5.1482	-1.7909	0.4657	-0.3978	1.7225	0	-2.2928
miR-291-3p	0.7655	-0.1488	0.541	0.1878		-6.6439	0.2154		1.2327	1.585	-2.2653
miR-344		2.1519	-4.6188	2.7866	1.3357	-0.8468	0.585	-6.6439	2.7549	0.2869	-2.2085
miR-187	-1	0.2412	-6.6439	1.8074		0.926		1.926	0.6781	-0.1297	-2.1949
miR-200a	1.2928	0.696	0.3219		-6.6439				1.2035	1	-2.1297
miR-302b	0.2987	-1.5437	-0.9296	0.2606	1.1603	-0.0544	1.3606	-1.5146	0.2339	-1.3959	-2.1241
hsa-mir-520c,hsa-mir-520b,hsa-mir-520f	-1.2969		1.9635	2.1043	-6.6439	0.0854		0.585	-0.3004	1.433	-2.07
miR-126	0.1916	-0.1552	-0.7013	0.3984	0.9501	1.2388	-1.9942	-0.8084	-0.226	-0.9296	-2.0358
miR-17-3p	1.585		-6.6439		-1.415	1.7442		1.926	0.8172	-0.0439	-2.0304
miR-211	0.0704	1.3785	0.2594	2.1375	-0.0233	-3.1844	-1.2413	1.5361		-2.9105	-1.9776
miR-350	0.1557		0.5018	-6.6439	-0.3346	2.5484	-0.2533	0.1375	-0.3165	2.2479	-1.957
	-0.2294	0.779	-0.7909	0.3896	-0.5249	0.5351	-0.9971	-0.1488	-0.1078	-0.7612	-1.8564
	0.1467	-0.304	0.308	0.4594	-0.3959	-1.0559	0.0014	-0.6416	-0.1219	-0.1456	-1.7494
miR-140	-0.1031	-0.9051	0.9351	-0.1361	-0.7588	-0.3789	-0.8339	-0.1062	0.926	-0.3201	-1.6811
miR-10b	-0.949	-1.8365	-0.0559	-1.221	1.3835	1.6055	0.2473	-0.8651	0.7181	-0.6016	-1.5747
miR-92	0.2522	-0.4286	-0.4093	-0.4901	0.4436	-0.8443	-0.9379	-0.6171	0.6727	0.7916	-1.5672
miR-196b	-0.9799	-3.7939	0.9389	0.3606	1.7506	0.6663	0.3651	0.3885	-1.4779	0.2265	-1.5552
miR-320	0.2666	0.0635	-0.0306	0.08	-0.0604	-0.097	-0.0087	0.0144	-0.8314	-0.8468	-1.4504
miR-434-5p	0.7137	0.7251	-1.7612	1.0314	-1.2042	-1.3255	0.7225	0.2016	-0.5084	0.103	-1.302
miR-23b	0.6239	-0.1313	-0.2987	-0.4679	0.263	1.3265	-0.4112	-0.2775	-1.071	-0.8262	-1.2704
	0.6835	-0.3511	-0.4286	0.0496	0.0663	0.0144	-0.1697	0	-0.3902	-0.699	-1.2248
miR-341	-0.1078	1.585		0.6323	1.406	-0.6371	-6.6439	0.6323	-0.0559	1.9819	-1.2072
miR-30b	-1.3959	0.3896	-1.0954	0.7049	0.7242	-0.6943	-0.2863	0.9479	0.9426	-1.3659	-1.1286

miR-30d	-0.7418	0.9613	0.099	-0.9856	0.45	-0.6552	0.4885	-1.074	0.0731	0.3115	-1.0732
miR-190	0.976	0.6323	-6.6439	0		1.5361		0	0.7049	1.7655	-1.0291
miR-345	1.9624	2.1043	0.1017	-0.5043	1.1699	-0.749	-0.1062	-6.6439	0.3951	1.4594	-0.8106
hsa-mir-339	0.269			1.0356		1.511	-5.088		1.5405		-0.7319
	1.0956	-0.9434	0.0759	-0.0755	0.1137	0.0129	0.4201	-0.1456	0.0029	-1.1456	-0.589
miR-380-5p	-3.872	-2.0893	2.2801	-0.0634	1.6088	-0.8084	-1.4739	1.7858	0.8464	1.2327	-0.5532
miR-144	0.3219	-0.4074	-0.3237	-0.1109	-0.0559	1.0983	0.4005	-0.2042	-0.3696	-0.7959	-0.4469
	-0.2845	-0.191	0.4772	-0.7515	-0.0801	-0.3585	0.3448	-0.7959	0.9811	0.2154	-0.443
miR-378	0.2091	0.3448	2.3735	1.9069	2.6439		-3.422	-6.6439	-0.4286	2.5969	-0.4194
miR-17-5p	-0.3328	-1.2176	-0.0145	-0.5735	-0.2176	0.992	0.338	-0.1187	0.6608	0.0718	-0.4121
miR-346	0.2702	-0.7612	0.545	1.8631	-0.0072	-1.3548	-0.5002	-0.3511	1.6332	-1.7226	-0.3856
miR-200b	0.6544	2.2327	2.3219		-6.6439	0.1043	2.4854	-0.852		-0.6597	-0.3569
miR-515-3p	1.3679	0.099	0.7225			1.1622	-6.6439	1.7225	1.4594	-0.2277	-0.3381
miR-15a	0.0745	0.1801	0.3196	-0.1456	-0.1234	0.0454	0.1283	-0.3827	-0.1877	-0.1779	-0.2694
miR-186			0.2522	1.5607	-6.6439	1.8679	1.8278	-0.3147		1.2016	-0.2484
miR-363	0.3918	0.9471	0.9583	-1.4659	1.0614	-0.2176	-0.46	0.9531	-0.725	-1.5479	-0.1047
miR-191	-0.5353	0.929	-0.2863	-0.2898	-0.5291	-0.1456	0.9672	0.3528	-0.4305	-0.0425	-0.0101
miR-184	-0.1109	-1.3111	-0.2176	1.4033	0.3606	1.0929	1.6599	0.5724	-2.4901	-0.949	0.0104
let-7a	-0.2934	0.7146	-0.8417	-0.6529	1.2516	0.2388	0.0676	-0.9771	0.539	0.0086	0.0551
	0.4552	0.4372	0.2339	-0.46	-1.2243	-0.5735	0.229	-0.3696	1.0236	0.308	0.0595
miR-302a	-1.7612	1.6323	0.8278	-0.4208	1.0356	1.6064	2.4594	0.8608	0.474	-6.6439	0.0704
miR-27a	-0.9574	0.1738	0.0841	-0.5907	-1.1203	0.472	0.2497	1.3248	0.8049	-0.3659	0.075
miR-101b	1.8679		-0.2379	1.848	1.433	2.3074	-1.5864		1.2016	-6.6439	0.1897
miR-195	0.4489	0.2904	0.0635	0.3863	0.0215	-0.8135	0.0412	-0.2934	0.1003	-0.038	0.2072
miR-352	1.1336	-2.8573	0.115	0.848	0.5341	-3.0469		0.7655	0.9635	1.7655	0.221
hsa-mir-425	0.1789	2.2129	-2.2311	-2.1584	-0.0291	-1.8625	0.3115	2.3528	0.9084	0.5568	0.2402
	-0.1472	0.9404	-0.6349	0.4813	0.1124	1.2546	-1.1392	-0.6484	0.1283	-0.0695	0.2778
miR-15b	1.1519	-1.1203	0.3885	-1.1329	-0.7784	-0.394	-0.125	-0.486	1.0996	1.6933	0.2967
miR-27b	-1.0619	0.2154	0.6799	0.809	-0.6552	0.6599	0.3346	-0.5416	-0.364	0.24	0.3161
miR-375		1.6088	-6.6439		1.406	1.6323	1.0704	-3.1203	2.1538	2.2327	0.3398
miR-224	-0.3165		-2.0116	-0.0499	-1.873	-0.2328	1.7004	-0.5564	2.6893	1	0.3495
	0.8875	0.103	0.0115	0.5301	-0.1047	-0.1959	-0.2294	-0.662	0.2066	-0.1361	0.4106
	1.1322	-0.2277	0.6808	-0.0291	0.2892	0.6663	0.4813	-0.7202	-1.0954	-0.6943	0.4831
miR-290	-1.4035		1.3219	2.2327	-6.6439	1.3219	1.3785	-0.5973	0.7225	2.1859	0.5187
	0.8528	-0.8186	-1.3771	0.3426	-0.1552	0.1674	0.6022	-0.6126	0.8319	0.7321	0.5655
miR-20b	-0.0218	-0.8863	1.1408	-0.4759	-0.041	1.0976	0.543	-1.2379	0.2904	0.1966	0.6055
let-7f	0.2054	0.852	-0.3076	-0.0544	-0.0679	-0.3165	-0.1828	-1.0145	0.7442	0.7715	0.6294
	0.545	0.2054	0.2546	0.294	0.0129	-0.0306	-0.9434	-0.1536	-0.2653	0.7824	0.7014
let-7c	1.5069	-0.3328	1.8949	0.7286	-0.881	-0.0893	-0.4779	0.6425	-2.071	-0.1697	0.7512
	-0.3292	-0.1633	0.3829	0.3606	0.6699	0.7991	-0.5332	-0.0725	0.1852	-0.5249	0.7746
miR-337	0.1738	-1.8997	2.3505	0.9199	1.7866	3.2172	-1.5312	-1.1877	-2.7859	-0.2379	0.8056
	-0.9296	-0.7686	-0.1313	0.2762	0.315	0.0314	1.1336	0.1532	0.8536	-0.1266	0.8069
	-0.7784	0.9899	0.451	0.3896	0.5281	0.8576	-0.8034	-0.0574	-0.0755	-0.6735	0.828
miR-200c	-2.4982	2.7115	0.3584	-3.357	-0.1219	0.8813	-1.3183	1.4179	1.972	0.8148	0.8605
miR-106a	-0.3004	0.584	0.1966	-0.1568	0.3403	0.1532	0.8608	0.6154	-1.2793	-0.0755	0.9383
miR-7	-6.6439			2.0179	1			0.5008	1.7004	2.3923	0.9675
miR-106a	0.0243	0.5646	-0.0189	-0.3419	0.2485	0.6599	-0.2619	-0.1552	0.4005	-0.0923	1.0276
miR-301	-0.5249	0.8844	2.0243	-1.0233	1.1057	-2.8573	-0.9462	-0.0529	2.6339	-0.1665	1.0772
miR-381	-0.6666	1.7527	-1.6897	-0.2059	0.6323	1.1859	1.585	0.0676	-1.889	0.315	1.0873

miR-374	2.1859	0.5965	1.9624	-1.9771	-0.3603	-2.7959	-1.0291	0.7672	0.0621	1.7799	1.1916
miR-151		0.263	-0.4739	0.9635	1.3219	0.7225	-1.776		0	0.3414	1.3624
miR-99b	-0.1109	0.3022	0.5079	0.4792	-0.3346	0.515	-0.1959	-0.0619	-0.2026	0.5291	1.4275
miR-335	0.4751	-0.771	0.7066	-0.9714	1.0888	0.6135	-0.3058	0.5079	0.0454	0.0649	1.454
miR-26a	-0.6804	0.1903	0.6699	0.294	0.184	0.4552	0.5782	0.315	0.2079	-0.749	1.4651
	0.5666	-0.274	0.5627	0.9833	-0.3733	-0.1031	-0.2159	0.688	0.1789	-0.4921	1.5211
miR-429	-2.7274	1.4594	0.6323	0.0314	2.0531	-0.1779	-1.5395	0.8875	0.0963	1.0704	1.7856
miR-30e-5p	1.5724	3.0339	-0.2916	2.2461	-4.0685	0.0704	-0.5002	-0.6529	1.0179	-0.5973	1.8302
	-0.2328	-0.1943	-0.2092	-0.4679	0.7841	0.115	-0.3364	0.6654	1.0433	0.7058	1.873
	-0.5606	0.4103	0.3254	0.3651	0.0144	0.9434	-0.4941	0.0745	0.6951	0.115	1.8885
miR-19a	0.4668	0.6727	0.6754	0.6069	0.4542	-0.2193	0.9107	-0.454	-0.6394	-0.5799	1.8941
miR-196a	0.7949	1.1043		-0.1172	1.0704	-0.5585	0.4594	-0.0454	2.121	-2.9105	1.9184
miR-329	-4.8954	1.8278	0.4875	2.081	0.3161			0.3807	1.7502		1.9479
miR-451	0.5539	-0.6506	0.0949	0.5878	0.2228	1.237	-0.394	0.3685	-0.3997	0.3346	1.9552
miR-130a	-0.5043	-0.1665	0.7587	-1.9714	1.2461	0.4636	-0.8314	1.4044	1.4885	0.1865	2.0742
miR-155	-1.4035	0.1814	0.6654	0.315	-1.4189	0.3696	0.9553	0.7849	0.2154	1.419	2.0836
hsa-mir-519a-2	-0.7084	-1.3511	0.6645	-0.1649	1.9819	-0.5649	1.8074		0.3219	0.157	2.1434
miR-20	0.1801		-0.8573	2.406	0.0676	-0.3094	-0.6171	2.0179	0.2654	-1	2.1532
miR-471	0.2461	0.5588	-1.0801	-0.9269	0.3651		1	-0.486	1.2382	1.3161	2.2313
mir-527	1.511	-0.5228	-2.4901	-0.4364		-0.7442	2.0222	-0.16	2.8278	0.2762	2.2837
miR-362		-0.2636					-0.881	1.5656	1.9069	0	2.3279
hsa-mir-423	-0.0336	0.827	-0.7108	-0.7155	0.24	0.2116	-0.0484	1.2951	1.1077	0.2302	2.4033
miR-223	1.6187	-0.4921	0.7706	-0.7588	-0.48	1.5286	-0.7322	-0.7226	0.6341	1.0426	2.4089
miR-30a-5p	1.3288	-0.5125	1.2382	2.6893	-0.3292	1.2016	-2.3659	-0.4739	0.2016	-0.55	2.428
miR-198		-1.6712	2.0875		-3.2379	1.6554	0.7163		0.3219	2.585	2.457
miR-32	-1.0679	0.1506	0.8695	-0.0801	0.0718	-1.7418	0.719	0.844	1.9305	0.903	2.5986
miR-336	2.991	0.1414	0.2726	0.1661	0.3242	0.7866	-0.5951		-0.1877	-1.0087	2.8904
	-0.3884	1.1276	0.5069	1.0996	0.5984	-0.1187	1.0264	0.0786	-0.0786	-0.8625	2.9893
miR-343	1	-0.2636	1.7655	1.3962	-2.4112		1.5351	-1.9051	-0.097	2	3.0199
miR-373	1.6323	1.3219		1.585	0.111				0.0201	-1.6394	3.0309
miR-213	2.1519	0.4049	2.1699	-6.6439	2.5729	-0.1016	-0.1282	0.7655	2.5236	-0.6016	3.1134
miR-16	0.6229	-0.6394	0.2738	0.5049	0.4542	0.6031	0.3033	0.5568	-0.415	0.8711	3.1357
	-0.1681	0.1801	-0.2898	-0.2176	-0.3111	0.8205	1.1197	0.9426	0.144	1.1104	3.3307
miR-19b	-0.1993	0.5597	0.251	1.2881	0.5753	0.9986	0.1362	0.6248	0.1349	-0.7322	3.6371
miR-129-3p	1.3516	0.9169	0.229	0.6826	0.6654	-1.1681	-0.2092	0.4147	0.4114	0.3516	3.6459
miR-330	0.5753	2.3329	0.6498	0.3311	-0.4016	1.858	1.585	2.1538	-6.6439	1.4201	3.8605
miR-134	-1.4659		-1.1203	2.121		1.7655			0.3752	2.2172	3.8927
miR-126	1.0172	0.189	-0.5395	-0.4266	0.3929	-0.0679	0.965	1.1878	0.9657	0.2558	3.9394
miR-215	0.669	0.1375	-0.7809	2.6206	1.4125	0.3219	-0.484	-1.077	0.3528	0.7807	3.9531
miR-133a	-1.255	0.1375	-0.1568	-0.0649	1.5361	1.2684		2.3074	-0.6193	0.8024	3.9558
	1.0656	0.1801	0.2857	-0.394	0.0813	1.0895	1.168	-1.5353	1.4152	0.6003	3.9564
miR-22	-6.6439	1.7442	1.9069	0.7225	2.3646	1.4854	1.5361	1.1375	0.1467		4.4
let-7g	-0.0072	-0.737	1.129	0.5763	0.0496	-0.2688	1.2887	1.1157	1.0724	0.9381	5.1568
miR-133b	2.6629	0.0759	1.6631	-0.6148	-1.6439	-2.5821	0.7416	0.7655	2.511	1.8074	5.3866
miR-28	-0.949		2.1699			-1.6897	1.6554	2.1375	0.433	2.0356	5.7927
miR-468	-0.5312	-0.2951	0.5811	0.7698	0.9411	-0.4679	0.6924	2.7225	2.281	-0.7226	5.9711
miR-297	2.2327	0.2558	1.6323			2.1375		0.4542	-0.3022		6.4103
miR-294	1.6088	0.8875		0.107	-1		1.4683	2.2172		1.1699	6.4587

hsa-mir-19a	0.8875	0.5361	1.433	0.2016	1	1.4854			1.6323		7.1759
miR-200a		-0.4901	1.1237	0.4916		2.2928	0.2016	0.4895	2.0704	1	7.1795
miR-299-3p	0.1699	2.0704	2	-1.2379	1.6708	0.8512	-1.2277	-1.8943	1.6699	3.121	7.1933
miR-125b	2.517	3.1859	-1.221	0.7268	2.0356	2.2928	-1.8836	1.8875	0.2179	0.2388	9.9977
miR-139	1.8875			2.6668	0.2892	0.2558	1.6699	2.2801	2.5607		11.61
miR-100	2.2479	-0.0499	0.6323	-1.6943	1.7004	2.6439	0.6257	2.9069	2.4931	1.9819	13.4879
miR-155	3.0515	2.5509	2.1437	2.1938	2.1483	2.9592	3.1431	2.0742	3.2472	2.4404	25.9523
miR-221	4.2365	3.0206	2.7879	3.4155	3.9495	4.1326	2.4668	3.1032	2.7058	3.0688	32.8872
miR-21	5.6929	8.1431	6.7879	7.47	6.5486	6.8354	8.2946	6.3029	8.2654	8.0806	72.4214
